# Comparison of Alveolar Bone Grafting Outcomes using CBCT in Individuals with UCLP Based on the Presurgical Orthodontic Treatment Methods

**DOI:** 10.1177/10556656221143945

**Published:** 2023-02-07

**Authors:** Jaemin Ko, Samantha Rustia, Lateefa Alkharafi, Rumpa Ganguly, Stephen L-K Yen, Snehlata Oberoi

**Affiliations:** 1 Craniofacial and Special Care Orthodontics, Division of Dentistry, Children's Hospital Los Angeles, Los Angeles, CA, USA; 2 School of Dentistry, University of California, San Francisco, CA, USA; 3 Program in Craniofacial Biology and Division of Craniofacial Anomalies, Department of Orofacial Sciences, University of California, San Francisco, CA, USA; 4 Oral and Maxillofacial Radiology, Department of Orofacial Sciences, University of California, San Francisco, CA, USA

**Keywords:** bone grafting, computerized tomography, orthodontics, tooth movement, bone regeneration

## Abstract

**Objective:**

The purpose is to evaluate outcomes of alveolar bone grafting based on the pre-grafting orthodontic preparation methods.

**Design:**

Retrospective analysis of individuals with unilateral cleft lip and palate.

**Subjects and Settings:**

28 individuals with non-syndromic UCLP from two craniofacial centers, 14 individuals each from XXXX and XXXX.

**Interventions:**

The alignment group underwent maxillary expansion with incisors alignment while the non-alignment group underwent only maxillary expansion for presurgical orthodontic preparation.

**Methods:**

Initial and post-surgical CBCT scans were compared to observe changes in angulation of the incisor adjacent to the cleft site, alveolar bony root coverage, and bone graft outcomes.

**Results:**

In the alignment group, the buccolingual rotation decreased by 32.35 degrees (*p* = .0002), the anteroposterior inclination increased by 14.01 degrees (*p* = .0004), and the mesiodistal angulation decreased by 17.88 degrees (*p* = .0001). Alveolar bony coverage did not change after bone graft in both groups, and no difference was observed between the groups. Chelsea scale showed satisfactory bone graft outcome (category A, C) in 12 cases (85.71%) in the alignment group and 11 cases (78.51%) in the non-alignment group. The volumetric measurement showed the alignment group had better bone fill of 69.85% versus 51.45% in the non-alignment group (*p* = .0495).

**Conclusions:**

Alveolar bony coverage on the tooth adjacent to cleft sites did not change with alveolar bone grafting surgery in either of the alignment and non-alignment group. Presurgical orthodontic alignment does not induce root exposure nor poorer bone grafting outcome.

## Introduction

Cleft lip and/or palate (CLP) is among the most common congenital anomalies of the craniofacial region.^1^ CLP often accompanies a cleft of the alveolus, which is a bony defect between the premaxilla and distal maxillary segment.^2^ Even though some cleft centers try to avoid alveolar bone grafting through early gingivoperiosteoplasty,^3–5^ reconstruction of the alveolus through alveolar bone grafting is currently the accepted standard of care.^6–8^

The goals of alveolar reconstruction are to facilitate eruption of permanent teeth adjacent to the cleft site with sound periodontal support, to create a barrier between the oral cavity and nasal cavity, to improve the morphology of the nose and alar base, and to build a sound foundation for future restoration.^9,10^

Since alveolar bone grafting was first introduced by Von Eiselsberg in 1901,^11^ numerous practitioners have tried to find the best method and timing of the surgery. Drachter (1914) used tibia and periosteum to reconstruct the alveolus at the early stage, which was called primary bone grafting. Secondary bone grafting by cancellous bone from iliac crest bone was introduced by Boyne in 1972,^12^ and it was widely accepted by numerous surgeons as the standard method for repairing the alveolus and showed satisfactory grafting outcomes.^13,14^ Some surgeons argue early secondary bone grafting at 4 to 7 years of age is beneficial,^15,16^ but this is still a subject of debate.^17^

Prior to secondary alveolar bone grafting, individuals with alveolar cleft often undergo orthodontic preparation. The key intervention is maxillary expansion, which can create sufficient space to allow for better access of bone graft placement, develop proper arch form, and correct transverse discrepancies.^18^ Depending on the relative amount of constriction between the anterior and posterior parts of the maxilla, either symmetric expanders or differential expanders are used.^19^

However, there is still disagreement about which type of presurgical orthodontic treatment should be performed. Some centers routinely perform limited orthodontic treatment to align permanent maxillary incisors along with orthodontic expansion^20^ while others perform only maxillary expansion^21^ or only alignment without the expansion.^22^ However, there is still no clear rationale for adopting the various methods.

Few studies compared bone grafting outcomes with and without presurgical orthodontic preparation,^22,23^ but no study aimed at comparing different methods of presurgical orthodontic treatment to our knowledge.

The purpose of this study is to evaluate radiographic outcomes of alveolar bone grafting based on the two different pre-grafting orthodontic preparation methods. Cone-beam computed tomography (CBCT) images from a group with presurgical maxillary expansion alone and a group with maxillary expansion along with anterior teeth alignment were evaluated and compared.

## Materials and Methods

### Subjects

The study was approved by the Institutional Review Board (IRB) of XXXX (#10-00564) and XXXX (#20-06610). The subject of this retrospective study consisted of 28 individuals (9 females, 19 males) with non-syndromic unilateral CLP from University of California, San Francisco and Children's Hospital Los Angeles who underwent secondary alveolar bone grafting with iliac crest bone. 14 patients (8 male, 6 female) from University of California, San Francisco (alignment group) underwent pre-alveolar bone grafting orthodontic preparation composed of maxillary expansion and incisors alignment and 14 patients (11 male, 3 female) from Children's Hospital Los Angeles (non-alignment group) underwent only maxillary expansion for presurgical orthodontic preparation. The sample size was calculated based on the preliminary data. CBCT scans from 10 patients showed that the mean alveolar cleft defect volume was 692.98 mm^3^ (219.81). Based on the assumption that a difference of 25% is considered significant,^23^ the minimum sample size was calculated 14 for each group with given power of 0.8 and alpha of 0.05.

Maxillary expansion was achieved by Hyrax rapid palatal expander, a fan-shaped expander, or Quad-helix depending on presurgical status. Hyrax rapid palatal expander was selected when patients had both anterior and posterior constriction whereas a fan-shaped expander or Quad-helix was utilized when patients needed anterior expansion without posterior expansion. The expansion was continued until ideal maxillary arch form was achieved and left in place for retention until they underwent alveolar bone graft.

In the alignment group, the straight wire appliance with 0.018-inch slot and MBT prescription was used in addition to the expansion before the surgery. 0.014-inch NiTi was used for the initial wire, followed by 0.016-inch and 0.017 × 0.017-inch NiTi wire. Whereas the expander was removed at the time of the surgery and the achieved expansion was maintained by the fixed appliance and wire in the alignment group, the expander was kept in the mouth after the surgery to stabilize the maxillary arch in the non-alignment group.

The average age when patients underwent alveolar bone grafting surgery was 9.86 years old in the alignment group and 9.93 years old in the non-alignment group. The exclusion criteria included individuals with incomplete alveolar cleft, syndromic CLP, a previous history of alveolar bone grafting or gingivoperiosteoplasty, and missing maxillary central incisors. Patient demographic data was shown in Table 1.

**Table 1.** Patient Demographic Data.

	Alignment group	Non-alignment group
Patient numbers	14	14
Sex	8 Male, 6 Female	11 Male, 3 Female
Ethnicity	7 Hispanic, 5 Asian, 2 White	11 Hispanic, 2 Asian, 1 White
Cleft side	4 Right, 10 Left	1 Right, 13 Left
Expander	4 Hyrax, 10 fan-shaped	4 Hyrax, 4 fan-shaped, 4 Quad-helix
Active expansion (days)	53.25 (15.02)	43.00 (25.25)
Age at T1 (years)	8.87 (1.10)	9.17 (1.96)
Grafted age (years)	9.86 (0.95)	9.93 (1.97)
T2 follow up (months)	8.00 (2.16)	9.19 (4.90)

### Cone-Beam Computed Tomography (CBCT) Evaluation

CBCT scans were obtained at two time points. The first scans (T1) were taken before pre-bone grafting orthodontic preparation and the second scans (T2) were acquired 6 months to 1 year after alveolar bone grafting. The average follow-up period for the second scans were 8.00 months after the surgery in the alignment group and 9.19 months in the non-alignment group.

**Figure 1.**
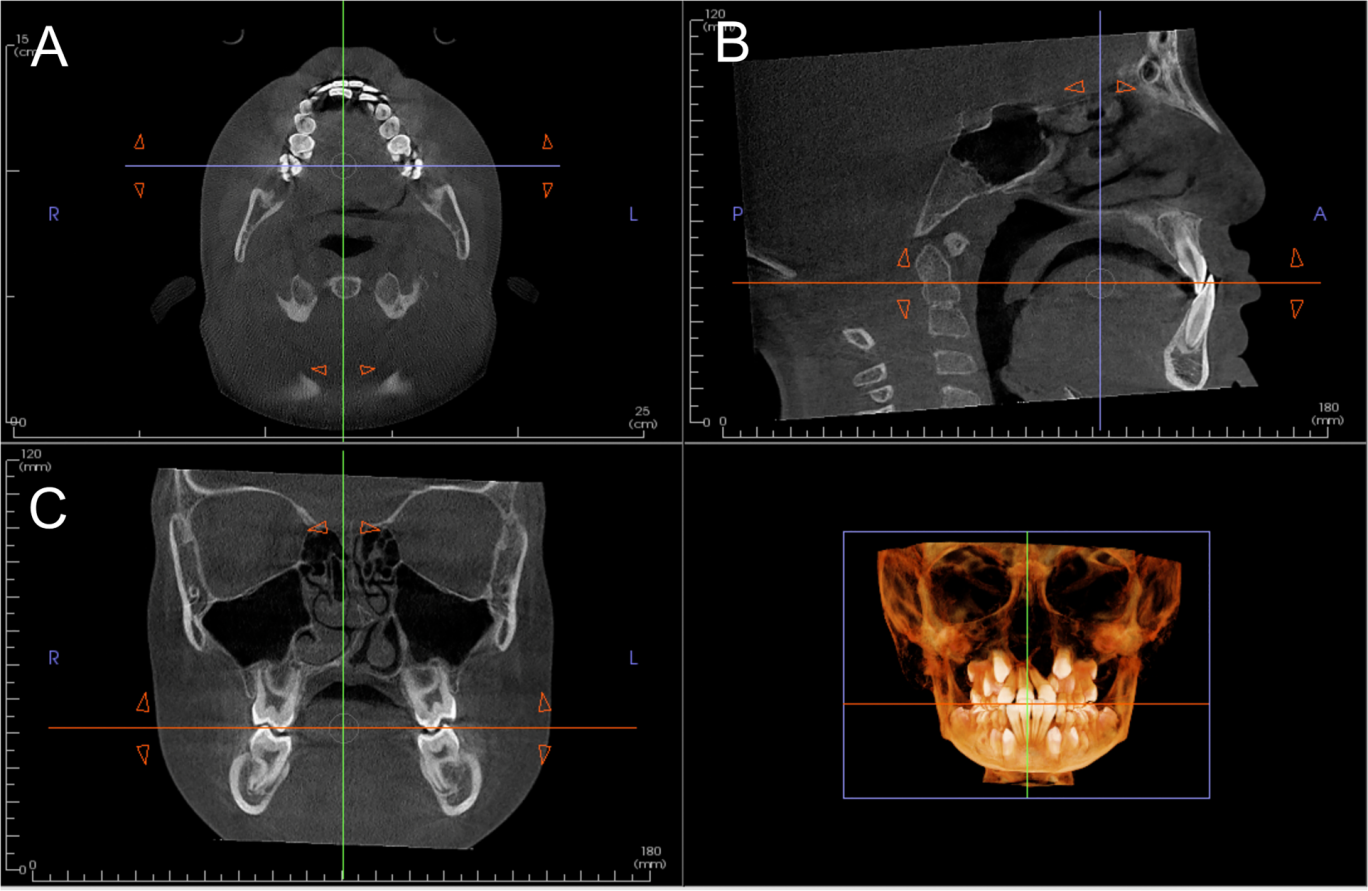
Orientation of the occlusal plane.

CBCT scans of this study were obtained separately from each craniofacial center, but the same x-ray machine model, CS9300 Cone Beam 3D imaging System (Carestream Dental, Atlanta, Ga) was used. The DICOM data of the CBCT images were exported to the imaging software InVivo (Anatomage, San Jose, CA). After data were loaded on the software, images were manually re-oriented. The occlusal plane was used as a reference plane and the axial plane, coronal plane, and sagittal plane were set accordingly. The occlusal plane was defined as a plane that connect overlap of central incisors and cusps of the right and left first molars. First, the palatal cusps of upper first molars on both sides were located using reconstructed volume and axial view. The axial view was set to show the both palatal cusps, and a yawing rotation was adjusted so that the both palatal cusps are on the same coronal plane (Figure 1A). Then, the axial plane was further adjusted with a pitch rotation so that the plane can pass through overlaps of upper and lower molar cusps and overlap of central incisors on the coronal view and the sagittal view (Figure 1B and C).

## Measurements

### Angulation Changes

**Figure 2.**
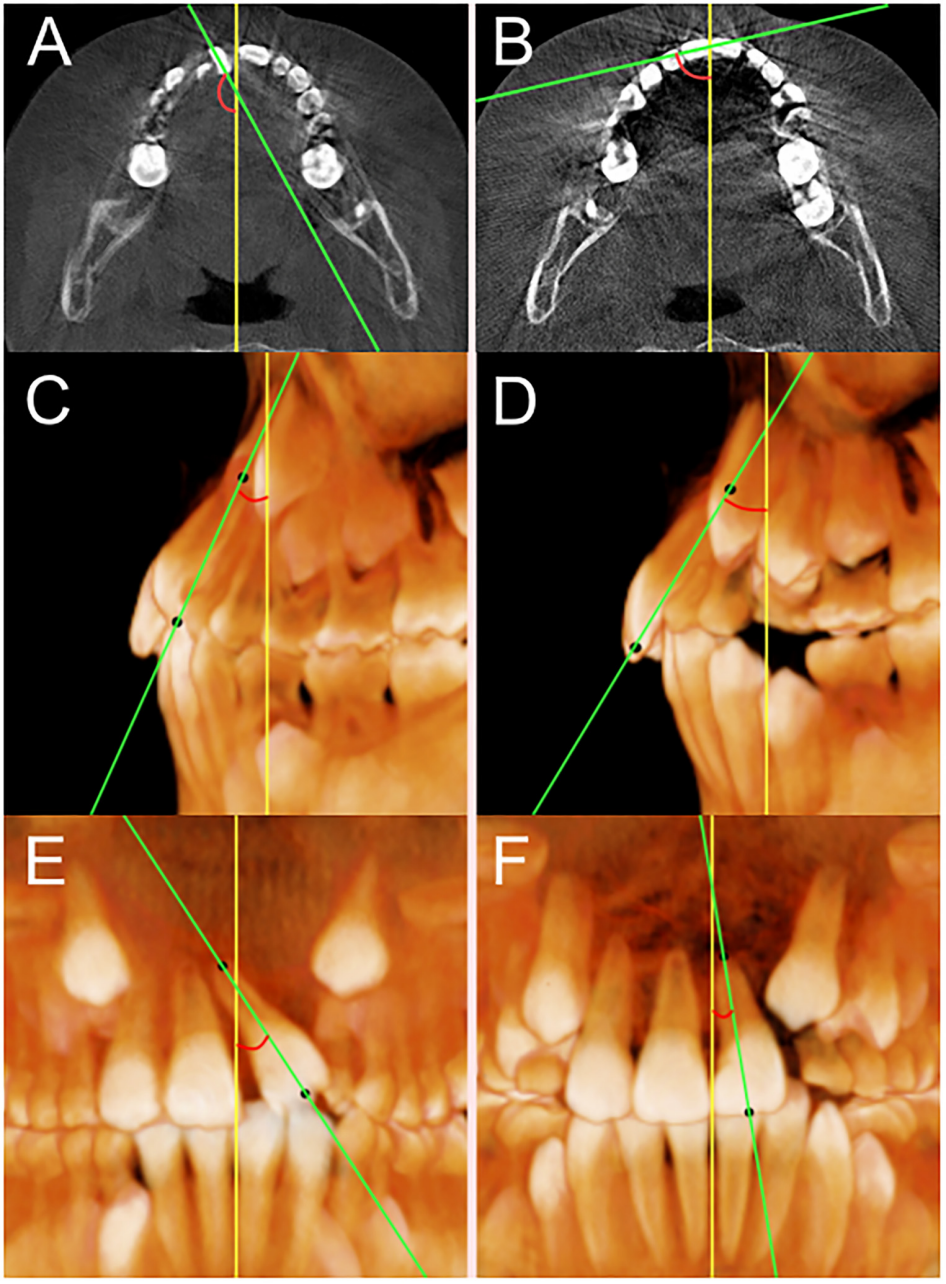
Measurement of angulation of the tooth adjacent to cleft site: A, buccolingual rotation at T1; B, buccolingual rotation at T2; C, anteroposterior inclination at T1; D, anteroposterior inclination at T2; E, mesiodistal tipping at T1; F, mesiodistal tipping at T2.

First, the changes in angulation of the incisor adjacent to the cleft site were evaluated in both groups. The angulation was measured in three different reference planes, which included the axial plane, coronal plane, and sagittal plane. In the axial plane, buccolingual rotation was measured. A line passing though the distal proximal surface and mesial proximal surface was drawn at the center of the crown (Figure 2A and B). Greater positive values referred to greater distal-out rotation. To measure anteroposterior inclination, the middle point of the incisal edge and the apex were positioned using a reconstructed three-dimensional (3D) image as well as axial, coronal, and sagittal slices. A line that connects the apex and the incisal point was drawn, and this line was projected to the sagittal reference plane. On the sagittal plane, the anteroposterior inclination was defined as the angle between the projected line and the coronal reference plane (Figure 2C and D). Greater positive anteroposterior inclination values meant greater proclination of the incisor. The same line that connected the apex and the incisal point was projected to the coronal reference plane and the angle between the projected line and the sagittal reference plane was defined as the mesiodistal angulation (Figure 2E and F). Smaller mesiodistal angulation values referred to straighter teeth axes. All angulations in the three reference planes were measured at T1 and T2 and compared within and between groups.

### Alveolar Bony Root Coverage

**Figure 3.**
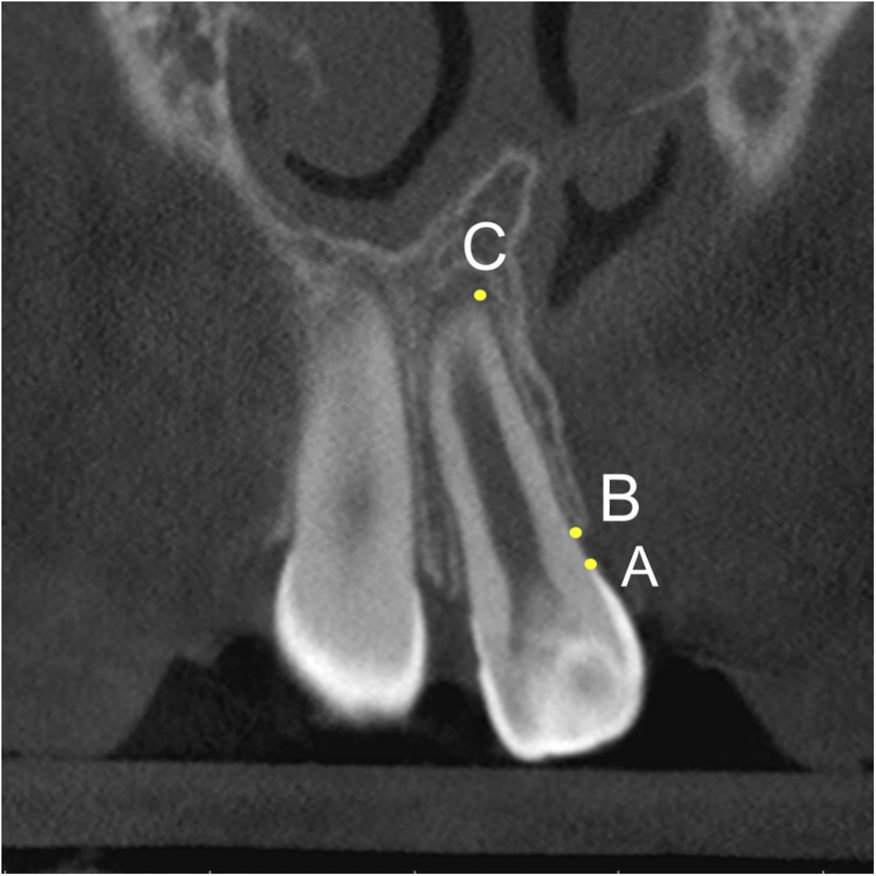
Measurement of alveolar root coverage: A, apex; B, alveolar crest; C, cementoenamel junction.

Second, alveolar bony root coverage on the tooth adjacent to the cleft site was measured. Before the measurement, the vertical reference plane was re-oriented so that the entire long axis of the tooth can be shown on the coronal slices. On the proximal surface closer to the cleft site, cementoenamel junction (CEJ, point A in Figure 3) and the point where the alveolar bony coverage starts to disappear (point B in Figure 3) were positioned. The distance between the apex (point C in Figure 3) and point A was measured as root length, and the distance between point B and point C was measured as alveolar bony coverage. The percentage of bony coverage to root length was calculated at T1 and T2 and compared within and between groups.

### 2-Dimensional Radiographic Evaluation

Third, 2-dimensional bone graft outcome was evaluated by Chelsea scale. Periapical radiographs were reconstructed from CBCT images. Dental arches were visualized on the axial sections and the arch area for reconstruction was manually defined so that the central incisor on the cleft site and the canine adjacent to the cleft site can be included in the periapical radiographs. Chelsea scale classifies the interseptal bony bridge into six categories: A. Bone must be present at the CEJ and at least 75% of both roots covered with bone; B. Bone must be present at the CEJ and at least 25% of both roots; C. Bone must be present across at least 75% of the cleft roots from an apical direction; D. Bone must be present across at least 50% of both roots from an apical to coronal direction; E. Any cleft site that has a bony bridge but does not have bone apically or coronally; F. Bone of 25% or less is present across both roots from an apical direction.^24^ After classification, category A or C was considered satisfactory bone graft, and category B, D, E, or F was considered unsatisfactory bone graft as proposed by previous studies.^25,26^

### Volumetric Measurements

**Figure 4.**
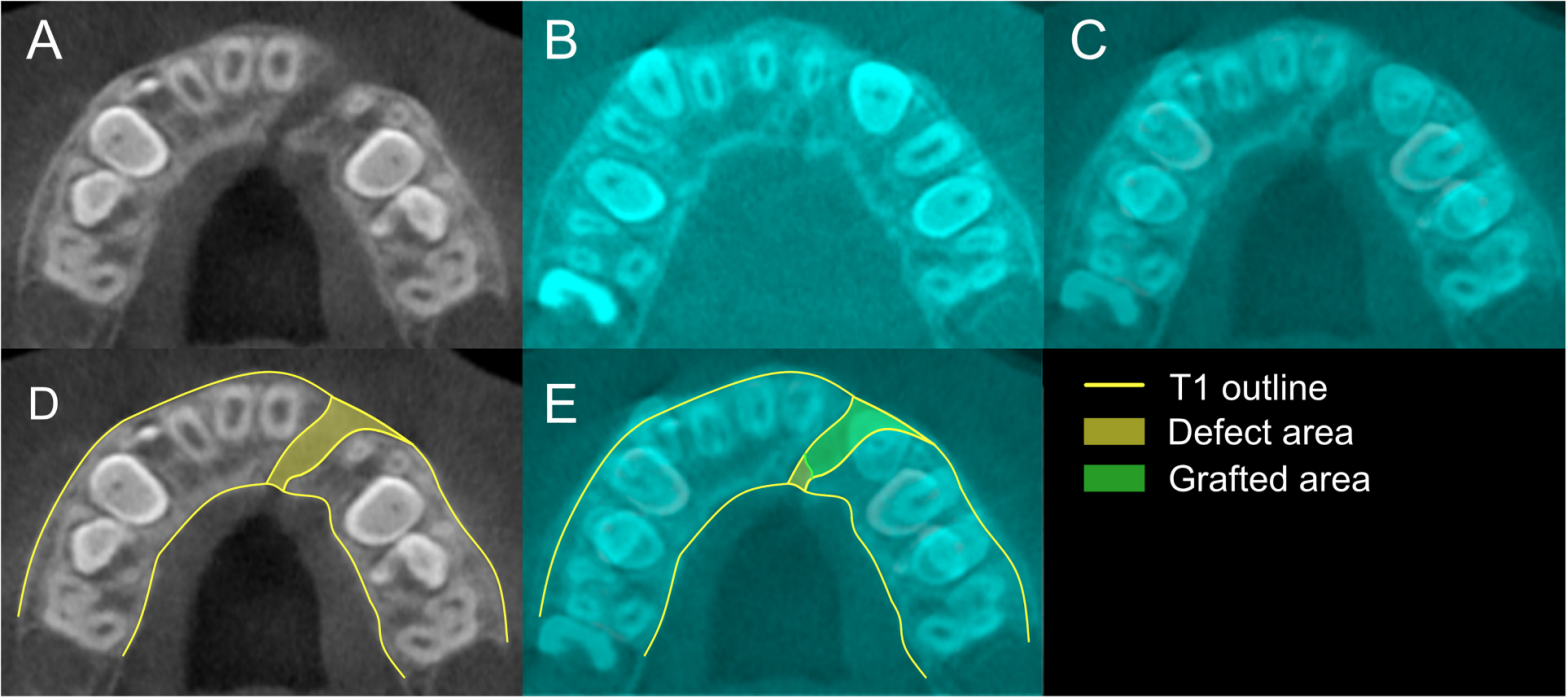
Measurement of graft fill: A, axial slice at T1; B, axial slice at the same position at T2; C, superimposed axial slices of T1 and T2; D, measurement of defect area at T1; E, measurement of graft filling area using outline drawn in figure D.

Lastly, the volumetric changes to the alveolar cleft were measured. The horizontal reference plane was re-oriented to the plane that passes the anterior nasal spine and posterior nasal spine so that the axial view can show alveolar bone more clearly while minimizing the showing of palatal bone. Then, the axial views of CBCT scans were observed on consecutive 0.5 mm slice sections. First, the pre-surgical cleft defect volume was calculated using the Cavalieri principle that consists of summing the defect areas of the axial slices and multiplying by 0.5 mm, the thickness of the axial slices. To measure the defect areas, the buccal and palatal contours of intact alveolar bone opposite to the cleft defect were used as a template for delineating the margins of the alveolar bone defect (Figure 4D). The boundaries of the cleft defect were measured from the CEJ to the apices of the teeth adjacent to the cleft site. Then, landmark-based 3D superimposition of T1 and T2 scans was utilized to visualize the volume of grafted bone (Figure 4C). The previously traced cleft defect margins were subsequently used to guide the tracing of the grafted bone (Figure 4E). The volumetric alveolar bone fill ratio was calculated as a percentage using the formula, the bone fill ratio = defect volume/grafted bony volume × 100.

The initial cleft defect was further divided into 4 layers depending on the vertical distance from the CEJ. The layers were delineated at 3 mm intervals from the CEJ. The first layer was from the CEJ to 3 mm apical to the CEJ. The second layer was 3 to 6 mm apical to the CEJ, the third layer was 6 to 9 mm apical to the CEJ, and the fourth and final layer was 9 mm apical to the CEJ and above. The initial defect volume and bony filling ratio was measured in each layer and compared between groups.

## Statistical Analyses

Statistical analyses were performed using STATA (StataCorp, College Station, TX). The mean measurements within each group were compared using paired Student t-tests, while the measurements between groups were assessed using unpaired Student t-tests. The 95% confidence intervals were used and the significance level was set to *p* ≤ .05. For angulation changes and volumetric measurement, multiple outcome analyses and subgroup analyses were performed to further investigate outcome. As multiple testing increases the risk of false positive result, *p*-values were tested for significance by Benjamini-Hochberg procedure.

To assess inter-rater and intra-rater reliability, two investigators each measured all of the T1 and T2 scans. The reliability of the measurements within and between raters was analyzed statistically using Cronbach's alpha test, with a kappa value ranging from 0.81 to 1.00 indicating “strong to near perfect” agreement.

## Results

### Angulation of the Tooth Adjacent to the Cleft Site

Table 2 shows the angulation changes in three reference planes in both groups. The buccolingual rotation statistically significantly decreased from 112.65 degrees at T1 to 80.30 degrees at T2 in the alignment group (*p* = .0002). In the non-alignment group, no statistically significant difference was observed between T1 and T2. When the buccolingual rotation angulations were compared between groups, T2 angulations were statistically significantly smaller in the alignment group (*p* = .0001).

**Table 2.** Changes in Angulation of the Incisor Adjacent to the Cleft Site.

	Alignment group	Non-alignment group	Difference	*P* value
T1 Buccolingual rotation	112.65 (26.46)	120.57 (28.21)	7.92 (10.73)	N/S
T2 Buccolingual rotation	80.30 (10.06)	118.19 (24.00)	27.89 (7.22)	.0001
Rotation changes from T1 to T2	−32.35 (6.28)	−2.38 (1.78)		
P value	0.0002	N/S		
T1 Anteroposterior inclination	8.46 (11.14)	4.81 (12.51)	−3.65 (4.64)	N/S
T2 Anteroposterior inclination	22.47 (9.72)	5.12 (12.76)	−17.35 (4.45)	.0007
Inclination changes from T1 to T2	14.00 (2.86)	0.31 (1.08)		
P value	0.0004	N/S		
T1 Mesiodistal angulation	27.99 (8.59)	20.26 (11.07)	7.74 (3.89)	N/S
T2 Mesiodistal angulation	10.11 (7.14)	20.18 (10.22)	10.07 (3.46)	.0076
Angulation changes from T1 to T2	−17.89 (3.05)	−0.08 (1.85)		
P value	.0001	N/S		

The anteroposterior inclination in the alignment group statistically significantly increased from 8.46 degrees at T1 to 22.47 degrees (*p* = .0004) at T2, whereas no difference was observed in the non-alignment group. T2 inclinations in the alignment group were statistically significantly greater than in the alignment group compared to the non-alignment group (*p* = .0007).

The mesiodistal angulation in the coronal plane statistically significantly decreased from 27.99 degrees at T1 to 10.11 degrees at T2 (*p* = .0001). In the non-alignment group, the angulations did not show any statistically significant difference. When the mesiodistal angulations were compared between the groups, the alignment group had statistically significant smaller angulation at T2 (*p* = .0076).

### Bony Coverage

Percentages of alveolar bony coverage on the tooth adjacent to the cleft site are shown in Table 3. At T1, alveolar bony coverage on the tooth adjacent to the cleft site was 89.47% in the alignment group, and 88.56% in the non-alignment group, which showed no statistically significant difference. At T2, the bony coverage was 85.63% in the alignment group and 87.38% in the non-alignment group, which also showed no statistically significant difference. When alveolar bony coverage percentages were compared between T1 and T2, no statistical difference was found in either group.

**Table 3.** Changes in Alveolar Root Coverage.

	Alignment group	Non-alignment group	Difference	*P* value
T1 Root coverage (%)	89.47 (5.89)	88.56 (6.00)	0.91 (2.29)	N/S
T2 Root coverage (%)	85.63 (10.84)	87.38 (7.33)	−1.75 (3.54)	N/S
Difference	3.84 (3.22)	1.18 (2.01)	2.66 (3.737)	N/S
P value	N/S	N/S		

### 2-Dimensional Radiographic Evaluation

Table 4 shows the Chelsea scale classification. Satisfactory bone graft outcome (category A, C) was observed in 12 cases (85.71%) in the alignment group, and 11 cases (78.51%) in the non-alignment group.

**Table 4.** Chelsea Scale Classification and Scores.

	Alignment group	Non-alignment group
A	7 (50%)	7 (50%)
B	1 (7.14%)	2 (14.29%)
C	5 (35.71%)	4 (28.51%)
D	0 (0%)	0 (0%)
E	1 (7.14%)	1 (7.14%)
F	0 (0%)	0 (0%)
Satisfactory	12 (85.71%)	11 (78.51%)
Unsatisfactory	2 (14.29%)	3 (28.43%)

### Volumetric Measurement

Table 5 shows the volumetric measurements of the cleft sites. The total volumetric size of the initial alveolar cleft defect at T1 was 683.08 mm^3^ in the alignment group and 773.88 mm^3^ in the non-alignment group, which showed no statistically significant difference. At T2, the alignment group had an average of 69.85% bone fill versus 51.45% bone fill in the non-alignment group. Statistical test showed that the alignment group had statistically significantly better bone fill (*p* = .0495).

**Table 5.** Initial Defect Volume and Bone Fill Ratio.

		Alignment group	Non-alignment group	*P* value
CEJ ∼ 3 mm	Initial defect (mm^3^)	122.37 (38.89)	145.95 (68.65)	N/S
	Bone fill ratio (%)	76.60	44.29	N/S
3 ∼ 6 mm	Initial defect (mm^3^)	158.58 (36.93)	183.87 (70.91)	N/S
	Bone fill ratio (%)	76.81	52.44	N/S
6 ∼ 9 mm	Initial defect (mm^3^)	213.61 (56.62)	226.33 (87.33)	N/S
	Bone fill ratio (%)	73.21	49.77	N/S
Above 9 mm	Initial defect (mm^3^)	203.03 (128.69)	234.47 (142.14)	N/S
	Bone fill ratio (%)	54.10	44.92	N/S
Total	Initial defect (mm^3^)	683.08 (141.31)	773.88 (265.76)	N/S
	Bone fill ratio (%)	69.38	48.00	.049*

## Discussion

Several studies reported that dental anomalies including missing teeth, supernumerary teeth, microdontia, malformed teeth, and positional anomalies are more prevalent in individuals with CLP.^27,28^ For the positional anomalies in particular, rotation of central incisors adjacent to the cleft site was reported in 25.7% to 77.14% of patients with CLP.^29,30^ Most previous studies that reported positional anomalies focused on observing rotation of the tooth adjacent to the cleft site, but few studies also observed other dental compensations including inclination and tipping of incisors and tipping of maxillary first molars.^28^

Our study showed that the tooth adjacent to the cleft site were distobuccally rotated, retroclined, and distally tipped prior to pre-grafting orthodontic treatment in both groups and corrected only in the alignment group. The degrees of angulation observed in the study were consistent with previous studies. Woods et al. compared the buccolingual rotation of the central incisor on the cleft side and the non-cleft side, and observed that the tooth on the non-cleft side was distopalatally rotated by 17.4 degrees from the coronal plane whereas the tooth on the cleft side was distobuccally rotated by 16.8 degrees.^28^ Chang et al. observed the decrease of buccolingual rotation from 41.43 to 10.54 degrees and mesiodistal tipping from 30.51 to 10.67 degrees with presurgical incisor alignment.^23^

Teeth adjacent to cleft sites have less alveolar bony support.^28,31,32^ Woods et al. observed that the cleft facing surface of the root had 5.7% less bony coverage compared to the contralateral tooth.^28^ Ercan et al. also reported that the tooth adjacent to the cleft had reduced bony support on the buccal surface.^32^ The initial alveolar bony coverage observed in our study was similar to the findings by Woods et al., who reported 83.1% of alveolar bone height support at the cleft site.^28^

Our bony coverage results suggested that that alveolar bone grafting does not increase nor decrease the bony coverage on the root at the cleft site. In other words, even when surgeons try to add bone grafting materials on the cementum surface over the alveolar bony coverage to make them bulkier, only pre-existing alveolar bone on the root takes the grafted bone. It can be interpreted that the grafted bone on the cementum surface was resorbed and could not create new bony coverage. It aligns with the core concept of the timing of secondary alveolar bone grafting, which is that bone grafting should happen before the canine surface is exposed to the cleft site.^13,14^

Excessive pressure from orthodontic appliances could push teeth out of the osseous envelope of the alveolar process causing dehiscence, fenestration, or vertical bone loss.^33–35^ Especially, this root exposure could be a concern for individuals with cleft as they have 0.6 to 2.8 mm of thin alveolar root coverage.^28^ However, the findings of bony coverage in this study showed that tooth alignment during presurgical preparation did not decrease the alveolar bone coverage on the root adjacent to the cleft site. Several previous studies also suggested that light orthodontic pressure against the alveolar bone housing does not necessarily cause root exposure out of the bony envelope since alveolar bone remodels as it creates new bony housing.^36–38^ It can be assumed that initial alignment of the anterior teeth by light force using NiTi wires does not push teeth out of the alveolar bone housing to the cleft defect.

CBCT has been recently used to evaluate initial bone defect and bone graft outcome. Whereas conventional two-dimensional evaluation methods only evaluate post-surgical images, volumetric assessment makes it possible to compare pre- and post-surgical images. Vig et al. mentioned that three-dimensional imaging is especially valuable tool in evaluating cleft palate,^39^ and numerous articles adopted volumetric assessment using CBCT to evaluate bone graft outcome.^15,40–42^ Measuring and calculating bone volume with CBCT is considered an accurate method to calculate the actual grafted bone volume as reported as reported by Shiroto et al. and Zhou et al.^43,44^

The volumetric measurement in this study showed that the mean volume of the initial cleft defect was 683.08 mm^3^ in the alignment group and 773.88 mm^3^ in the non-alignment group. Previous studies have reported various volumes for unilateral alveolar cleft from 610 to 1820 mm^2^, depending on the measuring method.^40,45^

The 69.85% of bone fill in the alignment group and 51.45% of bone fill in the non-alignment shown in our study corresponded to bone resorption rates of 30.15% and 48.55% respectively. The grafted bone undergoes an extensive remodeling and transformation process following alveolar bone graft surgery.^46^ With the remodeling process, some of the grafted bone at the cleft side undergoes resorption.^47,48^ A few different methods have been used to measure the bone resorption after alveolar bone graft. In a few previous studies, the volume of the initial cleft defect and the volume of the remaining grafted bone 1 year after the surgery were compared and bony resorption ratios between 16% and 51% were reported.^40,41,49^ Another way of observing bone resorption is directly comparing remaining grafted bone volume at different follow up time points. Nagashima et al. reported 48.8% of grafted bone volume resorption from 1 month post-surgery to 6 post-surgery.^50^

The Chelsea scale used in this study showed that the rates of satisfactory bone grafts were observed in 78.51% of cases in the alignment group and 85.71% in the non-alignment group. This was consistent with the previous studies which reported success rates from 72.5% to 86%.^25,26,51^ Even though both two-dimensional and volumetric measurement were performed in the current study, direct comparison of two methods was difficult due to inherent difference in measuring method. However, we noticed a tendency for the two-dimensional evaluation to overestimate the bone graft outcome. Many of cases that showed low bone fill with volumetric assessment were still classified as category A or C with Chelsea scale. lino et al. reported that bone graft outcomes were overestimated with two-dimensional imaging in approximately 40% of cases.^52^ A few previous studies performed direct comparison between two-dimensional and three-dimensional assessment. Rosenstein et al. reported that two-dimensional imaging overestimated graft outcome up to 21.4% and underestimated up to 17.7%.^53^ They concluded that three-dimensional evaluation is more accurate and reliable than two-dimensional evaluation. Han et al. explained limited accuracy of 2D assessment can be explained by structural overlap, distortion, and incapability of measuring buccopalatal thickness.^54^

It is hard to conclude that presurgical alignment leads to better bone grafting outcome due to the limitations of this study. The major limitation of this study was that some of the variables could not be controlled. As the alignment and the non-alignment group came from two different craniofacial centers respectively, the orthodontic and surgical providers were not identical. Though both centers used the same surgical methods using iliac crest alveolar bone, the fact that different surgeons performed the surgeries might have affected the grafting outcomes. Also, the composition of the expanders used in each group was not the same. Even though the goal of the expansion in all cases were the same, which was to restore the collapsed alveolar segments for better access for bone graft, different types of presurgical expansion might have affected the surgical outcome. However, this study is meaningful as it showed that presurgical alignment does not induce root exposure nor poorer bone grafting outcome. To understand the relationship between bone grafting outcomes and presurgical alignment, further studies are necessary.

## Conclusion

Alveolar bony coverage on the tooth adjacent to cleft sites did not change with alveolar bone grafting surgery in either of the alignment and non-alignment group. Presurgical orthodontic alignment does not induce root exposure nor poorer bone grafting outcome.
